# Reflections on an academic career

**DOI:** 10.1002/mgg3.298

**Published:** 2017-05-21

**Authors:** Judith G. Hall

**Affiliations:** ^1^ Departments of Medical Genetics and PediatricsUniversity of British Columbia and BC Children's Hospital VancouverBritish ColumbiaCanada

## Abstract

There are many undefined aspects to an academic medical career. This article attempts to provide some guidance about things to consider.

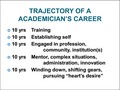

As I reflect on my academic career, I recognize several patterns, and some helpful insights emerge. Thus, I thought it might be useful to reflect in general on an academic career in the early 21st century. Mine has been in health sciences, but the concepts are potentially useful to all academic careers. However, the details may not all be applicable to other disciplines. Nevertheless, many of the general comments should be transferable.

I trained in medical/clinical genetics and pediatrics. Because I had been interested in clinical research related to congenital anomalies and nontraditional mechanisms of disease, my laboratory has been the clinic. I have been heavily engaged in medical genetic service delivery and training of clinical genetics fellows. I was head of two clinical genetics units for about 9 years each: one in Seattle, WA and the other in Vancouver, BC. I was the Chair of Pediatrics at the University of British Columbia (UBC) for 10 years. Mandatory retirement was still in effect when I reached the age of 65 years. I actually quite looked forward to retirement because it was possible to plan and set aside many projects to undertake after retirement.

## Changing Demographics

When I was born, my life expectancy was 59 years, but over the last 70 years, that has changed, such that my life expectancy is now in the late 80s because I am a woman on the West Coast of Canada. I have become part of the first group of academicians who can anticipate a life span with an extra 30 years. Giving some thought to one's overall career, including the last third, seemed to make sense.

In reflecting on my own experience, it would appear that about one‐third of my life was spent into preparation and training for a career in academic medicine. About one‐third of my life will have been spent “working” within an academic setting. And about one‐third of my life may well be spent utilizing my lifetime of experience in new ways. In other words, academicians today are pioneers of a new type of scholarly life because of the expanded life span. It comes with unique opportunities to utilize their acquired skills.

## Trajectory of an Academic Career

**Figure 1:**
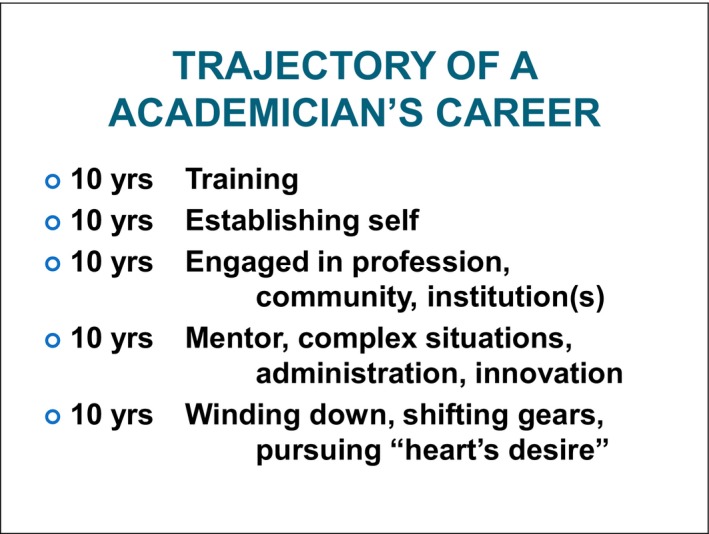
Typical Academic Career by decade.

Another way to look at the typical academic career is by decades (Figure 1). The first 10 years are aimed at very specific training: in my case medical school, residency, fellowship, and other kinds of specific training.

The second decade of an academic career is primarily spent establishing oneself in a chosen discipline, as well as perhaps within an institution and professional group.

The third decade is really about engaging in the profession, in the local community and in the institution(s) at which one is working.

The fourth decade is about utilizing one's experience: being a mentor, undertaking much more complex situations, providing administration, and introducing new techniques both in education and research. It includes preparing qualified professionals to continue in one's footsteps.

Finally, the last 10 years have been a kind of “winding down” and shifting gears – beginning to think about how to use all of that life experience, as well as pursuing one's heart's desires. Trying to see the “big picture” and prepare for an unpredictable future.

## Ten Career Changes Over a Lifetime

Many years ago, I remember hearing that most professionals have multiple career changes over their working life. More recently, there has been the suggestion of as many as 10 career changes are normal. In fact, the younger generation thinks that job changes are viewed as positive in that you have been exposed to a variety of different working worlds, and that is desirable to the employer. I remember when I first heard this that I thought that it would be unlikely for me, but in reflection, I am well aware that one moves from being a student, to a trainee, to a house officer at many different levels, to a fellow, and to a postdoctoral fellow. Once in the academic stream, from an instructor, to an assistant professor, to an associate professor, and full professor. But at the same time, one is an author, a reviewer, an editor, a researcher, and a grant writer. Once you are experienced, you become an adjudicator on committees, advisory boards, granting councils, and editorial boards that oversee the academic industry. Most individuals rise to being Chief of their clinical service or research unit, or, as Assistant Chief, taking on important capacities for their group. Many people become Chairs of Departments, Deans, or Head of institutions. None of that reflects their other more personal roles of being a wife/husband, mother/father, daughter/son, grandparent, household executive (housewife) and being engaged in many different ways in one's community.

## Learn the System at Your Place

There are three important systems to learn about, which are unique to the situation: (1) organizations (the organizational chart – who reports to who); (2) the kinds of resources available; and (3) how to get feedback and evaluation.

It is important to understand the organization at “your place.” This means the hospital, the research institute, the medical school, the university, and the community to which you belong. Understanding the systems and the rules (written and unwritten) of each system is really important to avoid conflict, to make regular progress in one's career, and to network between systems.

It is also important to understand what kinds of resources are available, whether they are automatic or competitive. What the range of available resources is – from information sharing, the availability of clinic time, access to trainees, travel money, etc.

The third major area that you want to learn about is how to get, and how often to get, feedback or evaluation. There will be someone to whom you report. They will have a boss and they are responsible for reporting about your performance. You need to make yearly written reports about the variety of your activities (at the same time as you update your CV).

You want to explore what types of additional opportunities are available within your institution: electives, grants, rotations, sabbaticals, exchanges, and awards (including monetary awards) so that you can take advantage of those in which you are interested or which will further what you are working on at the time.

As your career develops, there will be different expectations, both related to your level of expertise, publications, reports, and record, but usually there is the expectation that you should receive grants for support of your research.

It is worth understanding the relationships and obligations between students, trainees, house staff, and faculty or superiors, as well as the medical and research organizations around you. How do you get involved? Which are automatic and which are honorary? And what kinds of commitments do they require? How do you get nominated or apply?

Finally, there are differences between being in a small or large city, as well as being part of a small or large organization. Both provides opportunities, but can also confine your options. Thus, it is important to learn about the system in your unique situation – its advantages and limitations. This means that you have many opportunities available to you that you should make yourself aware of and may wish to pursue.

Choosing a variety of institutions at which to get your training provides a broader set of experiences to draw from later in your career. Professionals from all those experiences will become your colleagues, future resources, and collaborators.

## No Single Way

The more experience and exposure you have, the more you realize there is not a single model and not a single way to do things. Furthermore, each person is a unique individual so working out their own pattern, thinking about it, planning it, and trying different things out, is important.

## The Training Decade – First Decade

It goes without saying that choosing a place to train has lifelong ramifications, but perhaps the most important thing about your training experience is that you enjoy yourself and the people around you. I always learned the most from my co‐trainees as a medical student, as a house staff officer, as a fellow, and as a young faculty member. You want to identify all the special opportunities at your institution as compared with others places and take advantage of those from which you will benefit. However, you want to remain flexible and keep your options open so that you do not get into limited areas where there are few options. Meanwhile, you also want to be developing some type(s) of special expertise in a particular area so you become useful because of your knowledge (Figure 1).

There is an enormous role for serendipity – that is, finding something good without really looking for it. It comes from having a positive attitude and keeping your eyes open; learning from your mistakes and being willing to try new things (Hall 2003).

As you finish medical school or Ph.D training and head toward other training such as house staff and fellowships, it is important to do your homework – to look at the various advantages of the various places where you might train.

How many applications should you make? You always want to have a back up, but you want to be aiming for a place that fills your expectations – whether it is fantastic patient experience, a prestigious place to be from, or the best training you can possibly get.

You will almost surely go for an interview. Remember that you are actually interviewing that place to see if it is a place you want to go. Clearly, you want to think about the questions that you will be asked ahead of time and have sensible answers. They will want to know if you will fit in to their organization. You also want to have a set of your own questions that will help you decide if the organization is the right place for you.

### Your curriculum during training

You want to make sure that there are didactic components to your training, such as clinical rounds, journal club, unknown sessions, laboratory rotations, and technical training. You will be learning a great deal from the other trainees and so being in a situation where there are many other trainees is to your advantage. You want both clinical and laboratory experiences no matter where you are headed. If you are headed toward being a clinical researcher, you want to know what laboratory work involves, and how reliable it is. If you are headed toward a laboratory research career, you want to know what it takes to be a clinician and how you can collaborate with them. Either way, you want to learn how to collaborate and how to make agreements ahead of time to make collaborations work well.

### How many years of postdoctoral training are you expecting?

The usual is 3 to 4 years, but every additional year will almost surely provide you with some additional insights, techniques, or approaches that will be valuable. In many clinical programs, there will be documentation of your clinical experiences, and perhaps exams that reflect that you have conquered a certain amount of didactic material. It is useful to take as many examinations as possible, not only for the experience but also to have multiple certifications and initials after your name! All those letters after your name do make a difference at some point and you certainly do not want to have to go back later and take examinations.

### Mentors

There is a great deal of emphasis on finding the right mentor these days, but in fact you want different mentors for different things, at different times in your life, and for the different parts of your life. The person who models a great clinician may be very different from the person who models work/life balance. When you ask someone for their advice, they are usually quite flattered and they will then want to know how things have turned out. You can overdo it of course, but having different mentors for different parts of your life actually makes sense because no one person can do everything well. I also think it goes both ways. It helps that individual to know their advice was useful or that you have made a different decision than they suggested.

Part of your primary mentor/advisor's job is to look after your professional development. They should ask you to send in abstracts to various types of meetings, ask you to compete for various prizes, ask you to attend a variety of meetings – because those are all part of your professional development. And they should be nominating you for prizes and prestigious memberships.

You are actually doing something of an apprenticeship with your mentor. You are watching how they carry on their professional life. Because you may admire different aspects of different people, you may have several models for different parts of your professional life.

During your career development, you need to look after developing your CV (curriculum vitae) and document your various experiences carefully. You need to submit abstracts for meetings, you need to make some presentations at meetings, and you need to learn how to make good posters (because it may be your first introduction to your future colleagues). You can gain experience at your home institution during its annual research day and then go for regional or national meetings, and eventually international meetings. You need to get the experience of writing papers, and the many drafts that a good paper requires. You need to learn to select the right journal for your paper, respond to critiques, revise, and all the technical headaches of submission. Finally, you may actually have to ask to be considered for prizes such as the best poster, the best oral presentation, the best fellow, etc. – it does not necessarily happen spontaneously – you have to be nominated.

Writing papers is never easy, but it is a sign of an academician; and therefore, significant thought needs to be given as to how to organize the paper, whether you write one or two papers, which journal to consider, what the impact factors are, and whether that really matters. You need to look at articles in the journal to which you are submitting and make sure your article fits. Perhaps the best learning experience is when you fail. Reflect on why, what the comments of the editors were, if it is possible to redeem or rewrite the paper, or if you have clearly presented your research.

## Establishing Yourself – Second Decade

“Which job is really your first job?” can be an interesting question. As you move from being a postdoctoral fellow to an instructor, you need to give some thought to “the job.” You probably will be having an interview whether you stay at the same place or move. There needs to be an interview so you can understand what the expectations will be: how much time the clinic service will take, what your teaching assignments will be, how much preparation each will take, etc. Then you need to think about your research: what will be expected, number of grants and/or publications. Be realistic about what you can accomplish because you will be expected to be productive.

When you are choosing that first job, you want to be sure that you will be at a place where there are others with whom you will enjoy working; therefore, you actually need to be interviewing future colleagues and finding out what their expectations of you will be. You will need to have references (people who know you and your work), probably several, that say you are capable of the job you are taking on. So think about having different references for different things. Who are the right people from your past?

You will be expected to make some type of presentation if you go for an interview, so you might as well get together a good presentation “for the road.”

Wherever you go, you want to get an academic appointment so that you can be upwardly mobile. You need to learn the difference between clinical track and tenure track and what the expectations of those different tracks are. You also want to find out what kinds of other perks are available – whether you have to apply for them, ask for them, or whether they come automatically.

Questions that need to be answered in any job interview include the amount of salary, benefits, and retirement plan, but you may want to ask about that last. During an interview, you expect the institution to be competitive; and therefore, you need to know about other places. You need to know whether your income is guaranteed and for how long. Of course you want to know what kind of office and laboratory space are available to you. You want to know about resources such as IT, library, parking, travel money, (even football tickets), etc. You want to know whether or not laboratory costs for offsite testing are covered; whether publication, poster, and travel costs to meetings will be covered; you want to do some teaching because you want access to students, but you do not want it to be something that takes all of your time. So you need to be quite clear about teaching responsibilities and opportunities. Finally, you want there to be reasonable expectations in terms of numbers of patients, numbers of papers, numbers of grants, number of lectures, etc.

Before you arrive at your new job, you can think about the grants that you can put together ahead of arrival. You can prepare some lectures and think about other scholarly contributions you will be wanting to make during the first year. You need to learn about the system at your new institution and to whom you will report. And then there are the practical things that need to be ongoing: housing, bank account, child care, etc.

You do want some visibility once you arrive because you do want people to know who you are, who you could work with, and how you get trainees to work with you. You want to make a presentation at Grand Rounds or the research institute in your first year. You want the opportunity of student teaching. You want to be on some committees so you can meet people.

### Committees: The rule of three

It is important to be on committees as one of the responsibilities of being a member of the institution to which you are going. The Rule of Three means that you are on three committees: one related to your profession, one related to your institution, and one related to your community. For instance, within your local pediatric society, you want to get on one of the committees such as program, social issues, government policy, etc. Within your institution, both the university/medical school and the hospital/clinic, you want to get onto committees so you learn how their systems work. Finally, you want to be part of your community, city, or region. It may be on a foundation, the YWCA/YMCA, a community center, or your block organization. This is in order to meet neighbors, and understand the unspoken rules.

The Rule of Three is that you stay on the three committees for 3 years, and then either become Chair or go onto three other committees. You only become Chair if there is something *you* want to accomplish, not just because nobody else will be Chair, and then you will only stay Chair for only 3 years.

The Rule of Three is really to enable you to learn how systems work, to make a contribution, to meet other people, and to feel that you are becoming “part of things.” Every one of the committees will be a worthwhile experience, but can become tedious or too time consuming if you stay more than 3 years.

### The academic stool

I had always heard that the academic stool had three legs: education, research, and service (clinical for an MD, free tests for a laboratory person). People are often described as triple threats: that is to say that they are superb in all three areas. However, the reality is that the academic stool really has four legs because administration is one of the most important aspects of academia. No department or institution will function without good administration.

As you are establishing yourself in academia, you must continue to further your knowledge and demonstrate excellence (but not in all four areas). You probably already know in which areas you excel. In some ways, that is easy because in all four areas, one can do research, be innovative, and publish findings. The interesting part is that you are frequently doing three or four things at the same time. So if you are in clinic, you are usually seeing a patient, teaching a student and having that patient be part of an ongoing research project that you are undertaking. If you are in academics, you almost always have students/trainees around, while you are doing work. However, the really important aspect is that if you are in academics, you should be furthering knowledge as well as demonstrating and modeling excellence. Otherwise you should not be there, you should be in private practice or in a business.

### Promotion – rule of four

Within the academic world, one is expected to be “productive” and sometimes that seems quite impossible. It is not really “publish or perish” – there is an obligation to advance knowledge. Yet, there is a helpful rule of four. If you publish four papers a year, you cannot not be promoted. Publications are part of the prerequisite for promotion. That sounds a bit like a heretic statement because we know that the journals you publish in make a difference. However, if you get organized, you will almost surely publish more than four papers a year. One paper will be a case report together with a student in which he/she can gain experience and you act as their mentor. A second paper could be a review of the literature and a series, either a clinical series or laboratory series, done by a resident or fellow, in which they get the experience of putting a paper (and possibly a poster) together, and you are acting as their supervisor. The third paper is from your own research, which is the area that you are working on, right at that time, where you are primary author. The fourth paper is a collaboration: if you are clinician, in collaboration with a basic scientist; if you are a basic scientist, in collaboration with a clinician. Four papers a year, year after year, gets you promoted!

### Keeping lists

The reality is that you are usually doing several projects at once and you need to keep a list of possible ongoing or new projects: the ones you are involved in and the ones that are the next logical steps. So when a trainee comes along, you have “ready” projects to suggest and they are at the right level. Obviously, you have to redo your list every year.

It is important to sit down once a year and take stock. Usually, this should be a fairly formal exercise for yourself where you take the day off and go off by yourself to a quiet place. It can be on or near your birthday, on or near the 1st of the year, or on or near the beginning of a fall term. At that time, you update your CV, and you make or update the list of your projects and interests. You set some goals for the next year. You review last year's goals and see what you have accomplished. Every year you will find that you probably accomplished about half of the things you planned to do. The other half drop by the way, but you picked up three or four new things that are every bit as exciting. After you have gone through the review and assessment and updated your CV, you make a list for the next year. Then, you make a “report” to your “boss” or maybe you have several “bosses.” It is important to touch bases yearly (or even more often) in a formal way with the person to whom you report and who is actually responsible for your career development. Let them know what you have accomplished. You can do these in writing or at a meeting, but you keep the list from year to year so you can observe your progress and new areas of interest.

### Promotion

Your promotion relates to all four areas of the academic stool and you should list the accomplishments in each of those four areas when you redo your CV on a separate sheet (some, of course, overlap the legs of the academic stool). There are some unwritten rules about promotion that I have observed over the years. For an Instructor, teaching critiques are really important, but publishing is essential to move from Instructor to Assistant Professor. Usually, it requires five to ten peer reviewed papers. Chapters do not count – which is disappointing because they are a wonderful experience. Reviewing information, finding unique areas of interest, and becoming an expert of a particular area are very useful, but chapters do not count for promotion. Promotion is really based on successful research, presentations at national meetings, and recognition from one's peers. Promotion to Associate Professor usually requires 20–25 peer‐reviewed papers, often acceptance into an honorary group, and presentations at international meetings. Promotion to Full Professor, require 60–70 peer‐reviewed papers and possibly a book. Twenty of those papers should be first or senior author. One must be known internationally within your profession's specialty group and have made significant contributions to the institution, the profession, and/or the community in which you are involved.

## The Third Decade

The Third Decade involves establishing oneself and being truly engaged in the profession, the community, and the institutions that are appropriate for your work.

### Change

Change can be a verb, noun, or an adjective, but it is inevitable as “death and taxes.” It is part of being a living organism, and is part of any organization or job. Medicine, biomedical research and in my case, human medical genetics, have changed enormously and will continue to change over a career.

When I entered medical school and was being oriented, the Dean said that “only 10% of what you learn over the next 4 years of medical school will be true in 10 years. However, we are not sure which 10% it is!” It is absolutely essential to keep up with the changes. It can be frustrating and exciting.

There are usually opportunities as change occurs. It is important to keep a positive attitude and to be flexible as the times change. In “Composing a Life,” Mary Catherine Bateson Bateson (2010) talks about the need for being “extemporaneous” – responding to the situation, to continually redefine who you are and what your job is. One must expect discontinuities and to “roll with the punches” in order to make the changes, which are sure to occur, meaningful. Holding on to the past is futile!

### Get a life and keep a life

It is really hard to keep a work‐life balance. One of the ways to do it is to be sure to take an hour a week, time on every trip, and a day in every year for reflection – to have time for yourself, to think about yourself, your family, your friends, and your job, and to rebalance.

### Sabbaticals – The Sabbath – every 7 years

I am convinced that the ancients who invented a Sabbath and sabbaticals were right. Every 7 years, every single molecule in your body turns over. Your body's cells may remain, but there is change going on everywhere. It is important to take time for reflection and to get new skills and new perspectives at regular intervals. Sabbaticals are a time to think and learn and reflect. During a sabbatical, it is important to get rid of all routine activities; to expose yourself to new ideas, new routines, and new ways of thinking; and to not actually have specific tasks or projects. Although usually you have to write a précis of what you are planning to do during a sabbatical, you do not actually have to do it! It is best to go away for a sabbatical, but actually the important part is just getting away for the first month or possibly first 3 months so that people are not expecting you to be there or depending on you.

It takes at least 2 years to plan a sabbatical. You have to find the right place, decide what you want to learn about, and get permission from your institution/department. Financing becomes an issue depending on what your institutional support is. If you are going out of country, you need to set up a bank account and a credit card in the new country. Your sponsor will help you arrange housing (and schooling if children are coming with you). Once you “arrive,” it takes at least 3 months to stop thinking about the “old stuff” (I remember on one of my sabbaticals to Oxford in 1988–1989 that I realized I was still dreaming about things back in Vancouver for the first 3 months). You are trying to clear your brain and reflect on new things.

### Reflection

**Figure 2:**
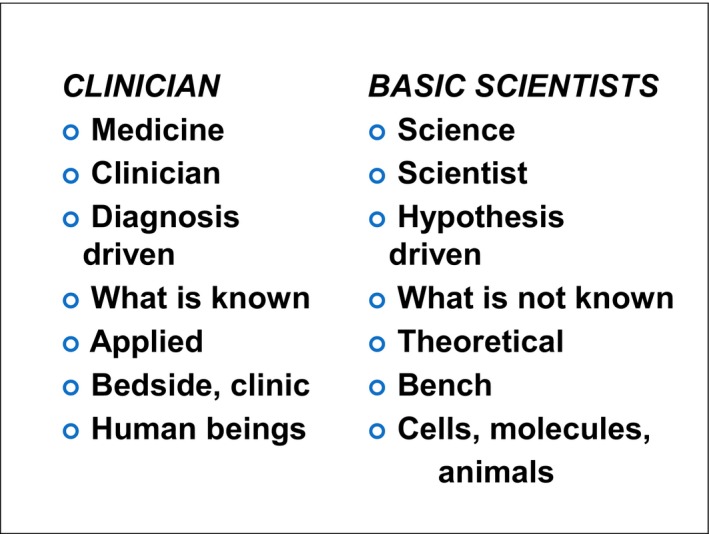
Comparison of Clinicians and Basic Scientists.

There is often a difference between how medicine and science approach the unknowns, although both take a scientific approach and both require asking the right questions in order to lead to changes in understanding and challenging the old ways of thinking. They require posing propositions/hypotheses and trying to answer them. When I was going through medical school, we really only thought in terms of objective data: make a hypothesis and collected the unbiased data to prove it. More recently, health researchers have developed methodologies that engage various types of subjective approaches as well. There are also those approaches where you engage the subjects in making the hypothesis (participatory research). Figure 2 compares clinicians and basic researchers. There is some truth to the comparison and the differences in approach; however, the best science is interdisciplinary. Clinicians deal with patients at the bedside, apply what is known, and make diagnoses. Basic scientists are hypothesis driven, trying to discover what is not known, and dealing with basic issues.

### Women professionals

**Figure 3:**
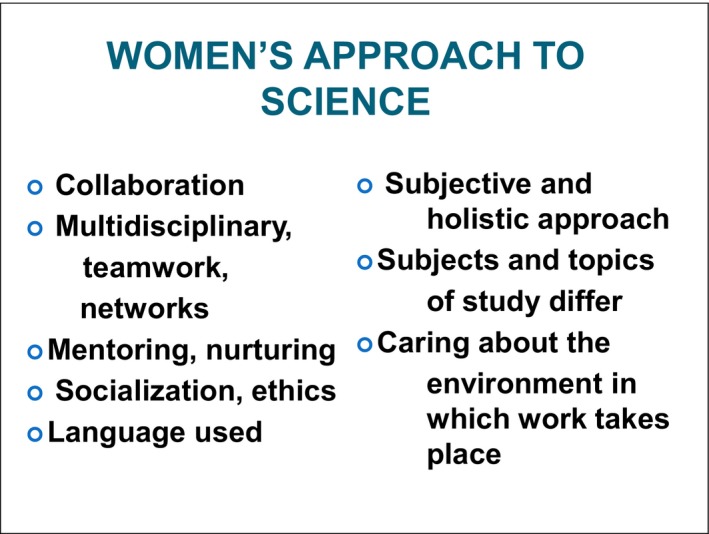
Frequent Characteristics of Women in Science.

The recent data is fairly remarkable about the number of women who are now in medicine and getting PhDs (UBC Association of Professors Emeriti). When I was a medical student, there were only five of us in a class of 75. Now 50% of medical students are women and this will probably change the profession. However, medicine is a natural profession for women to go into since it is related to caring and helping. However, only 10–15% of women are in administrative roles, although it is way past time for there to be many more (Hall 2005; Bateson 2010). One of the questions is why more women do not take up leadership roles. Probably, one of the major reasons is that the professional world has been built for males and it is extremely patriarchal in its approach. Secular work has been built on a model of competition, hierarchy, being completely engaged, and having a caretaker at home who takes care of the “home front.” But the “times are changing,” and real people are engaging in real professions and having real lives. Women often have a somewhat different approach (Figure 3) to science and medicine. They tend to be more collaborative and do multi‐disciplinary projects working in teams and networks. They tend to be nurturing and mentoring those around them, interested in ethical issues, and socialization. They often use a different language, take a subjective and holistic approach, care about the environments in which they work, and choose different topics for study than men might (which, in the past, have not been funded well).

My own view is that men's and women's approaches are complementary and biologically based. “Out of Africa” occurred only about 60,000 years ago and that means that our basic biology has not changed much. We are still very much governed by social mores which again, in almost every society, have been patriarchal, reinforcing the hunter‐gatherer male who is a warrior and a hunter, while women's role was raising children, socializing them, and providing food and homes. I recently heard a great quote having to do with when the men and women enter a room already filled with conversing people. The men are looking out for the alpha male to find out what is going on and whether he can be more alpha. The women go into the room looking for friends they know, networking and collaborating. I think it is important to keep those qualities in mind during an academic professional life. They are not at odds, but are actually quite complementary.

### Stress

You have chosen a stressful profession if you are in academic medicine and it is important to have ways to relieve that stress, but to also thrive on it. Some stress actually builds resiliency. It is important to learn from mistakes and move on. Challenges are often invigorating, but to put up with the stress, you have to have determination. It is called “fire in the belly”; the challenges and questions you are dealing with are so exciting that you cannot help, but propel yourself forward!

### The five goods

When I was in medical school, there was a female professor of pathology who took the five female students aside and said that we could come to her with questions. She said there were five things that helped her to continue to have a professional life. She called them the “five goods”:

1. *Good organization* – that is, you have to be an organized person with “to do” lists updated on a daily basis.
2. You have to have *good help* – you cannot possibly have a full professional life and be a full‐time housekeeper. I was fortunate enough to have a full‐time live‐in housekeeper from the time I had my first child in medical school. It just does not make sense to try to come home and do all things that need doing around a home after a day of work in the laboratory or the hospital. I wanted to come home and be with my children, but having a housekeeper does mean giving up a certain amount of control, valuing their help, paying them well, and letting them know how much they are appreciated.
3. A *good partner* or good friends for support – you will need people who really appreciate what you are trying to accomplish and with whom you can talk, let off steam and listen to their suggestions.
4. *Good Health* – that means both taking care of your health, but also being fortunate enough to have good health. The selection process that gets you to medical school suggests that you will have to have had good health, but that means maintaining it.
5. Last, but not least, is *good luck* – actually it is good fortune. Taking advantage of situations and making the most of them. Not encountering true disasters that throw you off your feet. You build your good fortune, in taking care of small problems, taking an optimistic/positive approach, reflecting regularly, and calling on those around you to make the situation work.

## The Fourth Decade

The Fourth Decade of an academic health sciences worker involves using your unique skills and the things that have brought you to your present situation and applying them to even more complicated situations. It means dealing with complexity and finding principles on which to interact. It means being innovative and finding new ways to meet the changing times. It means taking on some administration, either in your department, your institution, or your profession. It means extending your collaborations. It is an exciting time – that fourth decade. In this day and age, it means knowledge transfer, learning from situations, identifying principles, sharing them, and continuing to reflect and revise. This is the decade where you make your unique contributions, where you innovate, where you deal with complexity.

## The Fifth Decade

The Fifth Decade is one of winding down, shifting gears, the pursuing of your “heart's desire,” and starting to plan for retirement. Do we ever retire? I do not think so, but we need to do some planning for elder years, save money to live off of in a socially responsible way, and to find a replacement for ourselves (Graham et al. 1992; Vaillant 2002; Hall 2013; Spector et al. 2014).

We have done a study of what happens in academia (Hall 2003) and it turns out that one‐third of people retire the way our parents did – engaging in community and family, one‐third continue their scholarly academic pursuits, and one‐third take their skills and use them elsewhere in a different work setting with potentially a different work style and pace. In pediatrics, 45% of pediatricians continue to provide patient care after the age of 65. More men continue in traditional work styles, but the real principle is we need to find ways to help senior academicians plan for the later trajectory of their career because they probably have a good 30 years after their “retirement.”

My own experience was that when I became Chair of Pediatrics, 15 years before my own mandatory retirement, there were five people who would be retiring in the next year. I met with each of them and three of them were angry that they were forced to retire.

These three department members did not want to retire, but both the hospital and the university insisted on it, so they were unhappy. One other individual had planned very carefully. He came in and asked if the Department could type the last draft of the 35 papers he planned to write (there were no computers in those days). In fact, he did go off and write many of those, but he also took up painting at which he became very good. The fifth person (I know he would not mind my telling) had been a previous Chair of Pediatrics. He kept asking how he could be helpful. I finally suggested that he should write the history of the Department. He went off in a scholarly research mode and produced a magnificent book, and then went on to write several other historical books about the individuals at the university. He found his unique retirement niche.

In the present age of no mandatory retirement, it is actually quite a challenge to figure out *when to retire*. I found mandatory retirement helpful because you did not have to “lose face” and if you planned properly then you could do the things you wanted to after you stopped being paid.

All of that experience with retirees gave me pause, and I realize that senior academicians are actually an untapped resource. Their human capital has not been available in the past because of shorter life expectancy. So we should find new ways to use them. As a leader or Chair, it is important to think creatively about the unique talents of a specific individual. Starting at about age 55, faculty members need to think about their “last third” and the unique contributions they can make. However, this also requires flexibility and creativity within the ever challenging systems of academia and healthcare system, as well as the requirements of your university, research institution, and hospital, to use such people in the best ways.

### Retirement

An important question is whether one needs to be paid in “retirement.” There is a general feeling that you should not be doing the same thing you were paid to do without being paid. I actually think that this a patriarchal masculine view because if you plan properly, you will have plenty of income in “retirement” and you should be able to do whatever you want and not need to be paid. However, as times change, it is an important question about whether there is a tax incentive, a financial model for reimbursement for ongoing scholarly activities that allows one to continue to have appropriate income.

Retiring is actually complex (Graham et al. 1992; Hall 2005). You need to start to plan at least 10 years prior. In the old days this would have been at age 55. The tax laws make it appropriate to start to plan for sure at 60 and you need to have saved enough money to support your longer life span. A succession plan is one of the real responsibilities of academicians – be sure there is someone who is properly trained who can take over and see the “big picture” in your area. With regard to medical liability, you have to save your records for 21 years, and so some thought needs to be given to those records.

There are many things that change with “retirement” and you need to prepare for it (Vaillant 2002). The saddest people are those who have not developed a “life” outside of work. Because actually, social contacts, friends, family, and community become extremely important and keep one healthy. Health is a real issue and there are a bunch of common ailments for older adults, but maintaining good health requires systematically being careful about diet, exercise, and challenging intellectual pursuits. These are essential to maintain your health in order to age well.

There are a variety of new technologies and it is important to keep up with those if you expect to function in the mainstream. It is important to maintain contact, either through an emeritus group or an advisory role, with your institutions and with others in your profession. You may have the opportunity to have input into the institutions you love, both by donations of time and money. Being on various councils and advisory groups is fun and very useful because of experience and “institutional memory.” This will be a bit like the advice to those starting out: it is worth being on three committees for at least 3 years, but also to give up and rotate those committees so that you are not obstructing development. Your lifestyle will change as you retire and age, and so will your schedule.

The major commitment I made to retirement was that I would go for walk, have a good cup of coffee, read the paper, and do the Sudoku before I began any other work.

It is useful to think of the “retirement career” as a trajectory in 5 or 10 year cycles (Graham et al. 1992). As I have completed my first postretirement decade, I have celebrated it by taking a sabbatical from manuscript writing and invited presentations for a year. I now spend more time on research, travel, and on reflection. It is sort of a joke to say you take a “sabbatical from retirement”, but that is just exactly what I have done. Each academician will find their own way. And we have much to learn about the full trajectory of an academic career.

## Conflict of Interest

None declared.
